# New insights into Korea's trade relations: A comprehensive FNARDL analysis with 18 global partners

**DOI:** 10.1016/j.heliyon.2024.e39696

**Published:** 2024-10-22

**Authors:** Min-Joon Kim, Rianmahardhika Sahid Budiharseno, Tong Thanh Trung

**Affiliations:** a Department of Distribution Marketing, Catholic University of Pusan, South Korea; b School of Global Studies, Global College, Kyungsung University, South Korea; c Faculty of Fundamental Sciences, College of Technology, National Economics University, Viet Nam

**Keywords:** Correlation relationship, International trade, FNARDL model, Korea, Real exchange rate

## Abstract

This study applied the Fourier nonlinear autoregressive distributed lag (FNARDL) model to investigate the bilateral J-curve effect between Korea and 18 trading partners. As a result, negative asymmetric effects of changes in the real exchange rate were detected in some cases in the short run. However, in other cases, these effects were positive or exhibited both positive and negative trends. This variation stemmed from the heterogeneity of import and export products and the diverse reactions of exporters to exchange rate fluctuations. By contrast, positive asymmetric effects were evident in most cases in the long run. Through the Fourier function, the FNARDL model better captured the J-curve effect compared to the standard NARDL model. Furthermore, by using bilateral trade data, this study mitigated the problem of aggregate misalignment, compared to using a country's gross trade data with the rest of the world. Therefore, the hidden effects of the real exchange rate were unveiled. This study's findings will enhance policymakers' awareness of formulating exchange rate management strategies.

## Highlights

• Asymmetric impacts were comprehensively analyzed using the FNARDL model.
• Utilizing bilateral data had minimized the aggregate bias problem.
• Short-term impacts vary depending on the sum of partial series.
• Long-term impacts were predominantly positive; negative impacts were very weak.
• The Fourier function clearly showed the J curve between Korea and 18 trading partners.

## 1 Introduction

The relationship between exchange rates and trade balance has been a subject of great interest and debate among economists and policymakers worldwide. The exchange rate, which represents the value of a currency with reference to another, plays a crucial role in determining a country's international trade competitiveness and overall economic performance. Thus, understanding the impact of exchange rate fluctuations on the trade balance is essential for formulating effective monetary and trade policies, and predicting and managing economic crises.

Given this significance, understanding how exchange rate movements directly influence the trade balance is essential. The trade balance is the difference between a country's exports and imports of goods and services. A positive trade balance, or trade surplus, occurs when a country exports more than it imports, while a negative trade balance, or trade deficit, arises when a country imports more than it exports. The exchange rate, as a key determinant of the trade balance, influences the relative prices of goods and services between countries, affecting the competitiveness of domestic industries and the demand for imports [1,2].

Some studies have assumed that the impact of changes in the exchange rate is symmetric. A study analyzed the effects of counter-exchange rates for more than half of the United States' trading partners during 1975–2000 [3]. The trade activities of three Central and Eastern European countries with Germany were analyzed [4]. According to Ref. [5], the study investigated the foreign trade of Fiji and Moura, whereas [6] investigated Australia's bilateral trade. An autoregressive distributed lag (ARDL) model was proposed to investigate the response of Croatia's trade balance to currency depreciation [7]. Malaysia's bilateral trade balance with its 14 largest trading partners was investigated [8]. Based on [9], it evaluated the long-term effect of the real exchange rates between China and 18 major trading partners on China's trade balance, using a panel dataset for 2005–2009. The results revealed that the yuan's real appreciation had no long-term impact on China's trade balance. A study from Ref. [10], argued that the regression ratio in South Africa's manufacturing sector, using data from 1995 to 2010.

Moreover, a study evaluated the reciprocity ratio with Spain's tourism trade balance [11]. The results showed that the euro's depreciation increased tourist arrivals from Turkey, whereas its appreciation had no significant effect on Spain's tourism balance. Furthermore, other studies (e.g. Refs. [[12], [13], [14], [15]], and [16]) have argued that the impact of the exchange rate is proportional to the trade balance.

However, some publications have suggested that exchange rates may have a disproportionate effect on the trade balance. Based on [17], evaluated that the impact of counter-exchange rates on Turkey's bilateral trade with nine trading partners; the authors concluded that price reductions improve the trade balance but only in the long run. Some of Turkey's long-term trade relations are unstable, so the country cannot take advantage of devaluation to overcome its trade deficit with some trading partners. Likewise, a study evaluated South Africa's trade balance with four major trading partners, using data from 1994 to 2009 [18]. The study found that the trade balance did not necessarily improve when the rand was devaluated. Meanwhile, an asymmetric relationship between remittance ratios and the trade between 102 Mexican industries and the United States was demonstrated [19]. Another study [6], reported that the asymmetric impact of changes in the exchange rate on the UK's bilateral trade balance with eight trading partners. The study demonstrated that an increase in the pound's value had a different effect on the UK's trade balance than a decrease in its value from 1998 to 2015. Moreover, a recent study [20] clarified the asymmetric impact of counter-exchange rates on Vietnam's bilateral trade balance with the 27 EU member states and the UK.

The literature has extensively explored the response of Korea's trade flows to exchange rate fluctuations at the bilateral and industry/commodity levels. For instance Refs. [21,22], examined the impact of changes in the real exchange rate on Korea's trade with Japan and the United States, using commodity-level data, and found significantly different effects by industry. Moreover [23], assessed the effects of bilateral real exchange rate fluctuations between Korea and China on 33 trade sectors. Similarly [24], explored the impact of bilateral exchange rate movements on Korea's imports and exports with Vietnam, and found that bilateral real exchange rates had asymmetric effects on specific export and import sectors. In addition [25], examined trade between Korea and the United States and reported the positive long-term impact of exchange rate volatility on 24 of 69 industries. At the bilateral level [26], investigated the impact of real exchange rates on Korea's trade imbalances with major partners such as the United States and Japan. According to Ref. [27], analyzed that how the asymmetry of exchange rate changes affected Korea's bilateral trade balance with 14 economies. Additionally [28], discussed the broader economic impact of exchange rates on Korea's trade balance with 14 ASEAN countries, and argued that both macroeconomic stability and specific strategies are needed to mitigate the adverse effects of exchange rate fluctuations. These studies have often used linear ARDL or nonlinear ARDL (NARDL) models to capture the effects of changes in the exchange rates. However, these models have limitations in capturing smooth structural changes in time series data. In particular, because of the asymmetry and temporal instability caused by changes in the data structure, linear ARDL models are unsuitable for capturing the dynamics of economic processes [29,30]. Therefore, the potential impacts of exchange rates on trade flows remain unclear.

To overcome these limitations, the present study analyzed the short- and long-term impact of the exchange rate on Korea's bilateral trade balance with 18 partners (see Table S1), using the Fourier NARDL (FNARDL) model. Hence, the contributions of this study are unique. Unlike the traditional linear ARDL framework, the FNARDL model captures smooth structural changes in time series data and nonlinear effects. Furthermore, the Fourier function—used as an approximation—transforms the NARDL model to capture the asymmetric impact of exchange rate fluctuations on the trade balance, an approach rarely seen in previous studies in Korea. Therefore, our findings provide a more complete understanding of the J-curve phenomenon. In addition, this study introduces a comparative analysis with the NARDL model. The comparability of the FNARDL and NARDL models not only underscores the methodological advancements of the former but also demonstrates the differential effects of exchange rate movements in both short- and long-term scenarios. Our goal is to unravel the multifaceted dynamics of exchange rate variability. This study presents a comprehensive exploration that traverses theoretical underpinnings, methodological rigor, and empirical scrutiny. Advanced econometric models can be used to better understand the intricate interaction between exchange rates and trade balances as these models incorporate linear and nonlinear factors and asymmetric responses.

In addition, we broadened our analysis to include 18 of Korea's trading partners to gain a more comprehensive understanding of Korea's global trade interactions. This extensive dataset captures a wide array of bilateral trade relationships, allowing us to analyze Korea's trade balance across diverse economic contexts and trade dynamics. Using this inclusive approach, we aim to examine the J-curve effect within a more diversified and representative framework. This expanded scope not only uncovers patterns and insights that may be hidden in more limited studies but also provides valuable perspectives for policymakers and economists. By meticulously selecting these 18 countries, we aim to provide a more detailed and nuanced understanding of Korea's trade balance and responsiveness to exchange rate fluctuations, thereby offering valuable information for policymakers and economists in navigating the complexities of international trade.

The J-curve phenomenon is a well-documented concept in the international trade literature. This phenomenon was first studied empirically by Ref. [31], who described how a country's trade balance initially worsens after a currency depreciation before improving in the long run. According to Ref. [27], the J-curve effect provides critical insights into the short-term trade balance dynamics following exchange rate fluctuations; this knowledge is crucial for policymakers aiming to stabilize the economy during periods of currency volatility. An export-oriented economy like Korea would benefit from a deeper understanding of the J-curve phenomenon, which could inform timely exchange rate management policies to enhance trade competitiveness.

Therefore, this research paper aims to comprehensively analyze the impact of exchange rate fluctuations on the trade balance, considering various factors, theoretical frameworks, and empirical evidence. By examining the literature and conducting an in-depth analysis, this study seeks to shed light on the complex relationship between exchange rates and trade balance, exploring the mechanisms through which exchange rate movements affect a country's trade performance. Furthermore, this research will explore into empirical studies that have examined the impact of exchange rate fluctuations on the trade balance across different countries and periods. By doing so, we aim to identify common patterns and divergences, as well as potential factors that may explain variations in the relationship between exchange rates and the trade balance.

## 2 Methodology and data selection

### 2.1 Fourier nonlinear autoregressive distributed lag (FNARDL) model

The theoretical framework for the import-export demand function, developed by Ref. [32], considers the impact of exchange rates on trade flows at the bilateral level and is presented as Equations (1), (2):

(1) $$VX=VX\left(Y^{F},\;RER\right)$$

(2) $$VM=VM\left(Y^{D},\;RER\right)$$

Where VX (VM) is the value of the export (import) and $Y^{F}$ $\left(Y^{D}\right)$ is the real income of the trading partner (domestic). $RER=NER\times P^{F}/P^{D}$ is the real bilateral exchange rate, where NER is the bilateral nominal exchange rate of the currency of the trading partner per unit of domestic currency, and $P^{F}$ ($P^{D}$) is the trading partner's (domestic) price level.

To study the impact of bilateral exchange rate fluctuations on Korea's trade balance with partner i, we established the trade balance model by combining export and import demand functions, using the approach of some previous studies [20,33,34], as follows:

(3) $${LnTB}_{i,t}=\alpha +\beta_{1}Ln{GDP}_{t}^{KOR}+\beta_{2}{LnGDP}_{t}^{F}+\beta_{3}Ln{RER}_{i,t}+\varepsilon_{i,t}$$

Where ${TB}_{i,t}$ is Korea's trade balance with trading partner i, calculated as the ratio of Korea's exports to trading partner i to imports from trading partner i in time t. ${GDP}_{t}^{KOR}$ is Korea's real gross domestic product, representing Korea's real income. ${GDP}_{t}^{F}$ is the real gross domestic product of trading partner i, representing the real income of trading partner i. ${RER}_{i,t}$ represents the bilateral real exchange rate between Korea and its trading partner i. $\varepsilon_{i,t}$ is a white noise error term. According to the theory, when domestic income increases (decreases), imports of goods increase (decrease) because of the increased (decreased) demand for goods, improving the trade balance. Meanwhile, the increase in the domestic demand for goods resulting from higher foreign income boosts exports so the trade balance improves. Moreover, the real depreciation of the won increases exports but decreases imports, improving the trade balance. Thereby, we expect estimates of $\beta_{1\;}$ < 0, $\beta_{2\;}$ > 0, and $\beta_{3\;}$ > 0.

Equation (3) depicts the long-term relationship between the independent and dependent variables. Short-run dynamics should be included in Equation (3) to study the long-term effects in depth. The linear ARDL model developed by Ref. [35] is derived from Equation (3) and is represented by Equation (4) as follows:

$${\Delta LnTB}_{i,t}=\alpha +\beta_{j}Ln{TB}_{i,t-1}+\gamma_{j}{LnGDP}_{t-1}^{KOR}+\delta_{j}Ln{GDP}_{t-1}^{F}+\eta_{j}Ln{RER}_{i,t-1}$$

$$+\sum_{j=1}^{p}\rho_{j}{\Delta LnTB}_{i,t-j}+\sum_{j=0}^{q_{1}}\lambda_{j}{\Delta LnGDP}_{t-j}^{KOR}+\sum_{j=0}^{q_{2}}\varphi_{j}\Delta {LnGDP}_{t-j}^{F}$$

(4) $$+\sum_{j=0}^{q_{3}}\omega_{j}\Delta {LnRER}_{i,t-j}+\varepsilon_{i,t}$$

The ARDL model exhibits numerous advantages, including its ability to verify cointegration even when the variables in the model are in level form (I(0)) and/or first-difference form (I(1)). Additionally, the ARDL model remains effective even when applied to a small sample size or when the explanatory variables are endogenous [35,36]. However, exchange rate fluctuations can have symmetric or asymmetric effects on trade flows [[37], [38], [39]]. Furthermore, objective factors such as the 1997 Asian financial crisis, the 2008 financial crisis, and the COVID-19 pandemic may affect the data structure's breakdown [30]. Therefore, the linear ARDL model cannot scrutinize the asymmetric relationship between independent and dependent variables [40]. developed the NARDL model to capture nonlinearity between independent and dependent variables, unraveling complexity in time series datasets with numerous unknown structural breaks. The real exchange rate in the NARDL model is formulated by aggregating positive (${LnRER}_{i,t}^{+}$) and negative (${LnRER}_{i,t}^{-}$) fluctuations, denoted as POS and NEG, respectively. POS and NEG are calculated using the equations below:

(5a) $$POS={LnRER}_{i,t}^{+}=\sum_{k=1}^{t}{\Delta LnRER}_{k}^{+}=\sum_{k=1}^{t}max\left(\Delta Ln{RER}_{k},0\right)$$

(5b) $$NEG={LnRER}_{i,t}^{-}=\sum_{k=1}^{t}{\Delta LnRER}_{k}^{-}=\sum_{k=1}^{t}min\left(\Delta Ln{RER}_{k},0\right)$$

By combining Equations (5a), (5b) with Equation (4), the NARDL model is rewritten as follows:

$${\Delta LnTB}_{i,t}=\alpha +\beta_{j}Ln{TB}_{i,t-1}+\gamma_{j}{LnGDP}_{t-1}^{KOR}+\delta_{j}{LnGDP}_{t-1}^{F}+\eta_{j}{POS}_{i,t-1}+\theta_{j}{NEG}_{i,t-1}$$

$$+\sum_{j=1}^{p}\rho_{j}{\Delta LnTB}_{i,t-j}+\sum_{j=0}^{q_{1}}\lambda_{j}{\Delta LnGDP}_{t-j}^{KOR}+\sum_{j=0}^{q_{2}}\varphi_{j}\Delta {LnGDP}_{t-j}^{F}+\sum_{j=0}^{q_{3}}\omega_{j}\Delta {POS}_{i,t-j}$$

(6) $$+\sum_{j=0}^{q_{4}}\Phi_{j}\Delta {NEG}_{i,t-j}+\varepsilon_{i,t}$$

The long-term relationships in Equation (6) are represented by the coefficients $\beta_{j}$, $\gamma_{j}$, $\delta_{j}$, $\eta_{j}$, and $\theta_{j}$, while the short-term relationships are indicated by the coefficients $\rho_{j}$, $\lambda_{j}$, $\varphi_{j}$, $\omega_{j}$, and $\Phi_{j}$. Furthermore, Δ denotes the first difference operator. In addition, p, $q_{1}$, $q_{2}$, $q_{3}$, and $q_{4}$ are the lag orders chosen according to the Akaike information criterion (AIC). Although the NARDL model has replaced the traditional ARDL model to capture asymmetric effects, the NARDL model with a structural break assumes that the structural changes are known in advance and allows for sudden structural changes by capturing them with dummy variables. Nonetheless, time series often involve many unknown structural breaks [30].

We applied the FNARDL model to meticulously capture structural changes in the data and thoroughly analyze the relationship between bilateral real exchange rate fluctuations and Korea's trade balance with 18 economies. The FNARDL model is a modified version of the NARDL model that incorporates Fourier trigonometric terms to identify structural changes. The Fourier function does not necessitate prior information about the nature, frequency, or timing of structural breaks. In addition, unlike structural break dummies, the Fourier function does not require multiple parameters, thus offering adequate size and power properties [41]. Hence, the FNARDL model can be used to identify structural changes without the need to test or modify additional structural changes in the estimated model [42]. Therefore, the FNARDL model yields more robust experimental results than the standard NARDL model. The FNARDL model is expressed in Equation (7):

$${\Delta LnTB}_{i,t}=\alpha +d\left(t\right)+\beta_{j}Ln{TB}_{i,t-1}+\gamma_{j}{LnGDP}_{t-1}^{KOR}+\delta_{j}Ln{GDP}_{t-1}^{F}+\eta_{j}{POS}_{i,t-1}$$

$$+\theta_{j}{NEG}_{i,t-1}+\sum_{j=1}^{p}\rho_{j}{\Delta LnTB}_{i,t-j}+\sum_{j=0}^{q_{1}}\lambda_{j}{\Delta LnGDP}_{t-j}^{KOR}+\sum_{j=0}^{q_{2}}\varphi_{j}\Delta Ln{GDP}_{t-j}^{F}$$

(7) $$+\sum_{j=0}^{q_{3}}\omega_{j}\Delta {POS}_{i,t-j}+\sum_{j=0}^{q_{4}}\Phi_{j}\Delta {NEG}_{i,t-j}+\varepsilon_{i,t}$$

Where d(t) represents the Fourier function developed by Ref. [43], expressed as follows:

(8) $$d\left(t\right)=\sum_{k=1}^{n}\alpha_{k}\sin\left(\frac{2\pi kt}{T}\right)+\sum_{k=1}^{n}\beta_{k}\cos\left(\frac{2\pi kt}{T}\right)$$

[43] used the single frequency in Equation (8), proposed by Ref. [44].

(9) $$d\left(t\right)=\psi_{1}\sin\left(\frac{2\pi kt}{T}\right)+\psi_{2}\cos\left(\frac{2\pi kt}{T}\right)$$

In Equation (9), $\psi_{1}$ is the amplitude, $\psi_{2}$ is the displacement of the frequency component, k is the number of specific frequencies selected (Fourier frequency), t is the trend, T is the sample size, and π=3.14. Substituting Equation (9) into Equation (7) yields the complete FNARDL model, expressed as follows:

$${\Delta LnTB}_{i,t}=\alpha +\psi_{1}sin\left(\frac{2\pi kt}{T}\right)+\psi_{2}cos\left(\frac{2\pi kt}{T}\right)+\beta_{j}{LnTB}_{i,t-1}+\gamma_{j}Ln{GDP}_{t-1}^{KOR}$$

$$+\delta_{j}{LnGDP}_{t-1}^{F}+\eta_{j}{POS}_{i,t-1}+\theta_{j}{NEG}_{i,t-1}+\sum_{j=1}^{p}\rho_{j}{\Delta LnTB}_{i,t-j}+\sum_{j=0}^{q_{1}}\lambda_{j}{\Delta LnGDP}_{t-j}^{KOR}$$

(10) $$+\sum_{j=0}^{q_{2}}\varphi_{j}\Delta Ln{GDP}_{t-j}^{F}+\sum_{j=0}^{q_{3}}\omega_{j}\Delta {POS}_{i,t-j}+\sum_{j=0}^{q_{4}}\Phi_{j}\Delta {NEG}_{i,t-j}+\varepsilon_{i,t}$$

In Equation (10), the short-term combined impact of the previous trade balance on the current trade balance is calculated with $\rho_{j}^{*}=\sum_{j=1}^{p}\rho_{j}$; $\lambda_{j}$, $\varphi_{j}$, $\omega_{j}$, and $\Phi_{j}$ are the short-term dynamic parameters for Korea's gross domestic product, the gross domestic product of trading partner i, and positive and negative fluctuations in the bilateral real exchange rate of the Korean won with the trading partner's currency, respectively. The short-term combined impact of these parameters is calculated with $\lambda_{j}^{*}=\sum_{j=0}^{q_{1}}\lambda_{j}$, $\varphi_{j}^{*}=\sum_{j=0}^{q_{2}}\varphi_{j}$, $\omega_{j}^{*}=\sum_{j=0}^{q_{3}}\omega_{j}$, and $\Phi_{j}^{*}=\sum_{j=0}^{q_{4}}\Phi_{j}$. Likewise, γ, δ, η, θ are the corresponding long-term parameters, and their combined impact is calculated with $\gamma^{*}=\frac{\gamma }{\beta }$ , $\delta^{*}=\frac{\delta }{\beta }$ , $\eta^{*}=\frac{\eta }{\beta }$ , and $\theta^{*}=\frac{\theta }{\beta }$ .

In Equation (10), we consider all the values of k in the interval k = [0.1, 0.11, 0.12 … 3.99, 4.00], with an increment of 0.01. The value of k that results in the smallest sum of squared residuals is selected, using the F-test proposed by Refs. [35,40] to assess the cointegration among factors in the empirical model. Cointegration is confirmed if the F statistic exceeds the upper critical bound. By contrast, cointegration is rejected if the F statistic is smaller than the lower critical bound. However, establishing whether there is cointegration becomes challenging if the F statistic lies between the upper and lower critical bounds. Thus, the Wald test is performed as a final step to determine whether the real exchange rate has an asymmetric effect on the trade balance.

### 2.2 Data selection

This study uses the bilateral exchange rates of the Korean won with the currencies of 18 other countries from 1995 Q1 to 2023 Q2. The rationale for selecting 18 countries, rather than a narrower or broader selection, was to capture a wide spectrum of Korea's trading relationships, including different continents, economy sizes, and levels of economic development. This diverse sample allows for a more generalizable analysis of the J-curve effect, shedding light on both the commonalities and unique characteristics of a broad array of bilateral trade dynamics.

Data on nominal exchange rates were collected from the Korea Bank (KB). Data on exports and imports for Belgium, Austria, Spain, Finland, and New Zealand were gathered from KB and the Korea International Trade Association (KITA). Gross domestic product data were obtained from the Organisation for Economic Co-operation and Development (OECD). Finally, the price levels of Korea and its trading partners were proxied by the consumer price index (CPI). Data on the CPI were collected from the International Monetary Fund-International Financial Statistics (IMF-IFS) and Statistics Singapore in the case of Singapore.

## 3 Empirical results

### 3.1 Results of the unit root test and cointegration test

To apply the ARDL framework, the variables used in the empirical model, including dependent and independent variables, should be integrated of order zero (I(0)) or/and order one (I(1)), without any variables being second-order integrated (I(2)). To satisfy this condition, we assessed the stationarity of the variables using the ADF test, and the results are presented in Table 1. None of the variables used in the empirical model were I(2). Cases where the null hypothesis could not be rejected at I(0) suggest that the dataset had a unit root (i.e., I(0)). In the remaining cases, where the null hypothesis was rejected at I(1), all variables were stationary at their first difference (i.e., I(1)), implying that the variables were suitable for implementing the FNARDL model.

**Table 1:** Unit root test.

		ADF test		Perron test	
		Level	First difference	Level	First difference
AUS	TB	−4.94∗∗∗	−9.36∗∗∗	−6.45∗∗∗ [2017 Q2]	−11.42∗∗∗ [2017 Q2]
	${GDP}^{F}$	−1.70	−3.79∗∗∗	−3.67 [2013 Q4]	−5.52∗∗ [2011 Q3]
	POS	−2.29	−11.42∗∗∗	−2.93 [2000 Q3]	−12.10∗∗∗ [2009 Q2]
	NEG	−1.66	−10.24∗∗∗	−3.10 [1999 Q3]	−10.85∗∗∗ [2000 Q1]
AUT	TB	−3.47∗∗	−10.53∗∗∗	−4.33 [2010 Q2]	−12.92∗∗∗ [2014 Q2]
	${GDP}^{F}$	−2.11	−5.78∗∗∗	−3.23 [2004 Q2]	−5.44∗∗ [2007 Q4]
	POS	−1.76	−10.63∗∗∗	−23.63∗∗∗ [2001 Q4]	−53.26∗∗∗ [2002 Q1]
	NEG	−2.59∗	−3.63∗∗∗	−2.74 [2018 Q2]	−11.27∗∗∗ [2010 Q1]
BEL	TB	−4.76∗∗∗	−10.64∗∗∗	−5.93∗∗∗ [2010 Q1]	−14.56∗∗∗ [2009 Q3]
	${GDP}^{F}$	−2.06	−5.63∗∗∗	−3.40 [2002 Q2]	−33.54∗∗∗ [2009 Q4]
	POS	−1.76	−10.61∗∗∗	−32.55∗∗∗ [2001 Q4]	−75.67∗∗∗ [2002 Q1]
	NEG	−2.63∗	−3.60∗∗∗	−2.78 [2018 Q2]	−4.96∗ [2001 Q2]
CAN	TB	−4.72∗∗∗	−8.79∗∗∗	−4.82 [1999 Q3]	−11.17∗∗∗ [2005 Q3]
	${GDP}^{F}$	−1.75	−5.59∗∗∗	−3.68 [2002 Q2]	−5.19∗∗ [2009 Q1]
	POS	−1.37	−11.05∗∗∗	−3.58 [2011 Q3]	−11.32∗∗∗ [2008 Q3]
	NEG	−0.52	−11.87∗∗∗	−3.05 [2009 Q1]	−12.03∗∗∗ [2012 Q3]
CHE	TB	−2.90∗∗	−18.23∗∗∗	−4.27 [2006 Q3]	−20.20∗∗∗ [2015 Q2]
	${GDP}^{F}$	−0.59	−5.90∗∗∗	−5.90∗∗ [2006 Q3]	−17.01∗∗∗ [2009 Q1]
	POS	−1.68	−10.96∗∗∗	−2.74 [2003 Q3]	−11.65∗∗∗ [2008 Q1]
	NEG	−2.94∗∗	−4.17∗∗∗	−2.70 [2018 Q1]	−5.53∗∗ [1999 Q4]
CHN	TB	−1.98	−11.93∗∗∗	−3.84 [2018 Q3]	−6.57∗∗∗ [2008 Q4]
	${GDP}^{F}$	−1.65	−3.45∗∗	−1.76 [2004 Q1]	−12.53∗∗∗ [1999 Q4]
	POS	−1.71	−10.49∗∗∗	−3.60 [2007 Q4]	−11.05∗∗∗ [2008 Q1]
	NEG	−0.97	−11.45∗∗∗	−3.10 [2002 Q1]	−12.03∗∗∗ [2009 Q3]
DEU	TB	−1.49	−16.09∗∗∗	−4.11 [2010 Q2]	−16.54∗∗∗[2011Q3]
	${GDP}^{F}$	0.43	−5.58∗∗∗	−3.93 [2015 Q2]	−6.22∗∗∗[2009Q2]
	POS	−1.99	−10.89∗∗∗	−5.96∗∗∗ [2001 Q1]	−17.05∗∗∗[2002Q1]
	NEG	−2.51	−3.67∗∗∗	−2.70 [2018 Q2]	−11.32∗∗∗[2010Q1]
ESP	TB	−1.84	−10.50∗∗∗	−4.17 [2008 Q1]	−12.52∗∗∗ [1999 Q3]
	${GDP}^{F}$	−2.42	−4.22∗∗∗	−3.01 [2010 Q4]	−5.25∗∗ [2007 Q4]
	POS	−2.26	−10.80∗∗∗	−4.60 [2001 Q4]	−14.34∗∗∗ [2002 Q1]
	NEG	−2.23	−3.91∗∗∗	−2.69 [2017 Q1]	−7.38∗∗∗ [2010 Q1]
FIN	TB	−2.35	−11.55∗∗∗	−4.31 [2008 Q4]	−12.08∗∗∗ [2012 Q4]
	${GDP}^{F}$	−3.22∗∗	−3.09∗∗	−3.24 [2003 Q2]	−21.11∗∗∗ [2009 Q1]
	POS	−1.83	−10.67∗∗∗	−15.63∗∗∗ [2001 Q1]	−37.69∗∗∗ [2002 Q1]
	NEG	−2.60∗	−3.81∗∗∗	−2.54 [2018 Q2]	−11.33∗∗∗ [2010 Q1]
FRA	TB	−3.01∗∗	−17.53∗∗∗	−5.74∗∗ [2011 Q1]	−18.28∗∗∗ [2011 Q1]
	${GDP}^{F}$	−2.62∗	−5.23∗∗∗	3.06 [2003 Q3]	−18.15∗∗∗ [2008 Q3]
	POS	−1.83	−10.65∗∗∗	−16.45∗∗∗ [2001 Q1]	−38.94∗∗∗ [2002 Q1]
	NEG	−2.30	−3.71∗∗∗	−2.75 [2018 Q2]	−11.27∗∗∗ [2010 Q1]
GBR	TB	−3.43∗∗	−15.32∗∗∗	−6.95∗∗∗ [2011 Q2]	−7.39∗∗∗ [2012 Q2]
	${GDP}^{F}$	−2.33	−11.66∗∗∗	−2.75 [2002 Q2]	−13.06∗∗∗ [2009 Q2]
	POS	−1.87	−11.43∗∗∗	−3.14 [2010 Q2]	−11.87∗∗∗ [1999 Q2]
	NEG	−1.23	−3.85∗∗∗	−3.37 [2018 Q4]	−6.76∗∗∗ [2008 Q3]
IDN	TB	−2.49	−12.18∗∗∗	−4.59 [2010 Q3]	−7.45∗∗∗ [2006 Q1]
	${GDP}^{F}$	−0.41	−5.96∗∗∗	−3.60 [2007 Q1]	−9.11∗∗∗ [2003 Q4]
	POS	−1.94	−9.52∗∗∗	−2.22 [2010 Q2]	−9.93∗∗∗ [1999 Q2]
	NEG	−2.62∗	−7.80∗∗∗	−3.52 [2007 Q2]	−6.52∗∗∗ [1999 Q4]
ITA	TB	−3.35∗∗	−3.78∗∗∗	−5.09∗∗∗ [2011 Q1]	−4.94∗∗∗ [2000 Q2]
	${GDP}^{F}$	−2.57	−4.67∗∗∗	−2.75 [2000 Q2]	−5.40∗∗ [2013 Q1]
	POS	−1.86	−10.62∗∗∗	−24.51∗∗∗ [2001 Q1]	−61.10∗∗∗ [2002 Q1]
	NEG	−2.10	−3.71∗∗∗	−2.77 [2018 Q2]	−11.01∗∗∗ [2010 Q1]
JPN	TB	−2.77∗	−11.34∗∗∗	−4.22 [2001 Q2]	−11.61∗∗∗ [2001 Q3]
	${GDP}^{F}$	−3.15∗∗	−4.86∗∗∗	−4.85 [2008 Q1]	−5.83∗∗ [2009 Q1]
	POS	−1.34	−10.60∗∗∗	−3.54 [2007 Q4]	−11.79∗∗∗ [2008 Q4]
	NEG	−0.57	−3.81∗∗∗	−4.07 [2018 Q3]	−6.20∗∗∗ [2012 Q4]
NLD	TB	−2.56	−11.17∗∗∗	−4.81 [2014 Q3]	−17.36∗∗∗ [2008 Q4]
	${GDP}^{F}$	−1.62	−3.14∗∗	−4.22 [2011 Q1]	−5.41∗∗ [2014 Q3]
	POS	−1.96	−10.81∗∗∗	−7.02∗∗∗ [2001 Q1]	−18.73∗∗∗ [2002 Q1]
	NEG	−2.46	−3.76∗∗∗	−2.72 [2018 Q2]	−11.45∗∗∗ [2010 Q1]
NZL	TB	−1.78	−10.71∗∗∗	−6.89∗∗∗ [2002 Q3]	−8.54∗∗∗ [2007 Q4]
	${GDP}^{F}$	−0.63	−3.32∗∗	−3.85 [2008 Q1]	−7.15∗∗∗ [2013 Q2]
	POS	−1.82	−12.12∗∗∗	−3.50 [2000 Q3]	−13.05∗∗∗ [2000 Q4]
	NEG	−1.88	−10.24∗∗∗	−2.50 [1999 Q2]	−11.55∗∗∗ [2000 Q3]
GSB	TB	−3.08∗∗	−12.13∗∗∗	−3.89 [2006 Q1]	−12.43∗∗∗ [2011 Q2]
	${GDP}^{F}$	−1.13	−5.71∗∗∗	−6.39∗∗∗ [2005 Q3]	−5.31∗∗ [2003 Q2]
	POS	−1.14	−10.39∗∗∗	−3.57 [2007 Q3]	−10.78∗∗∗ [2008 Q1]
	NEG	−2.13	−10.85∗∗∗	−2.61 [2001 Q2]	−11.56∗∗∗ [2005 Q1]
USA	TB	−2.75∗	−7.74∗∗∗	−3.79 [1999 Q2]	−12.82∗∗∗ [2018 Q1]
	${GDP}^{F}$	−1.26	−3.19∗∗	−4.52 [2008 Q1]	−5.26∗∗ [2011 Q4]
	POS	−0.74	−10.56∗∗∗	−3.19 [2004 Q2]	−10.82∗∗∗ [2001 Q2]
	NEG	−0.35	−12.07∗∗∗	−3.99 [2018Q2]	−12.46∗∗∗ [2009 Q3]
Other variables	${GDP}^{KOR}$	−1.75	−3.68∗∗∗	−5.19∗[2001 Q4]	−6.00∗∗ [2002 Q4]

Note: 1) AUS: Australia; AUT: Austria; BEL: Belgium; CAN: Canada; CHE: Switzerland; CHN: China; DEU: Germany; ESP: Spain; FIN: Finland; FRA: France; GBR: United Kingdom; IDN: Indonesia; ITA: Italy; JPN: Japan; NLD: Netherlands; NZL: New Zealand; SGB: Singapore; USA: United States.

2) The ADF test stands for the augmented Dickey-Fuller test, whereas PP refers to the Phillips-Perron test.

3) ∗∗∗, ∗∗, and ∗ denote significance levels of 1 %, 5 %, and 10 %, respectively.

Table 2 reports the results of the cointegration test. In the NARDL model, the results did not support the cases of CAN, CHN, DEU, and the USA because the F-test values were below the lower critical bound (I(0)). Moreover, the F-test value was between I(0) and I(1) in the case of ESP and FRA. By contrast, in the FNARDL model, the null hypothesis (no long-term relationship between the variables) was rejected when the F-test value exceeded the upper critical bound (I(1)). This implies that cointegration existed in most cases (18 countries, respectively).

**Table 2:** Cointegration test results.

Model	Select k	AUS	AUT	BEL	CAN	CHE	CHN	DEU	ESP	FIN	FRA	GBR	IDN	ITA	JPN	NLD	NZL	SGB	USA
NARDL F-statistic	k = 4	3.51∗∗	5.87∗∗∗	10.76∗∗∗	1.99	4.42∗∗∗	1.75	1.41	2.29#	3.51∗∗	2.46#	8.76∗∗∗	3.51∗∗	5.91∗∗∗	3.67∗∗	3.89∗∗	8.33∗∗∗	4.53∗∗∗	1.46
FNARDL F-statistic	k = 4	5.23∗∗∗	11.87∗∗∗	12.26∗∗∗	10.01∗∗∗	6.52∗∗∗	5.74∗∗∗	4.95∗∗∗	3.14∗	7.52∗∗∗	5.03∗∗∗	9.68∗∗∗	14.08∗∗∗	7.95∗∗∗	3.70∗∗	15.32∗∗∗	9.02∗∗∗	10.81∗∗∗	5.35∗∗∗
	Critical bounds			10 %		5 %		1 %											
				I(0)	I(1)	I(0)	I(1)	I(0)	I(1)										
	F-Bounds test (k = 4)			2.2	3.09	2.56	3.49	3.29	4.37										

Note: 1) AUS: Australia; AUT: Austria; BEL: Belgium; CAN: Canada; CHE: Switzerland; CHN: China; DEU: Germany; ESP: Spain; FIN: Finland; FRA: France; GBR: United Kingdom; IDN: Indonesia; ITA: Italy; JPN: Japan; NLD: Netherlands; NZL: New Zealand; SGB: Singapore; USA: United States.

2) I(0) and I(1) represent the lower and upper critical bounds, respectively.

3) # represents the p-value falling between I(0) and I(1) at the 10 % significance level.

4) ∗∗∗, ∗∗, and ∗ denote significance levels of 1 %, 5 %, and 10 %, respectively.

### 3.2 Experimental results

We used quarterly data from 1995 Q1 to 2023 Q2 to apply Equation (10) and test the J-curve. We applied Equation (10) using the FNARDL model and the NARDL model to compare the results. The results of the F-test for cointegration are highly sensitive to the number of lags imposed on each first-difference variable [45,46]; thus, the number of lags cannot be imposed arbitrarily [27]. We selected a maximum lag of 4 for the dependent variable and regressors. AIC was used to choose the optimal model. The short-term coefficient estimates for variables with a lag of 0 were missing; thus, we added an additional lag of 1 for the POS and/or NEG variables in some models to obtain meaningful parameter estimates when considering the J-curve phenomenon. Table 3, Table 4 provide an overview of the short-term impact, long-term impact, and diagnostic test results in the NARDL and FNARDL models. We omitted the ${GDP}^{KOR}$ and ${GDP}^{F}$ factors for simplicity in understanding the short-term dynamic results and reported only the coefficients obtained for POS and NEG and the estimate of the lag error correction term (ECM).

**Table 3:** Results of the NARDL model.

	Trading partners																	
	AUS	AUT	BEL	CAN	CHE	CHN	DEU	ESP	FIN	FRA	GBR	IDN	ITA	JPN	NLD	NZL	SGB	USA
**Short-term (Panel A)**																		

Δ ${POS}_{t}$	0.625	0.253∗∗∗	−0.025	0.500	1.357	0.298∗∗	0.188	0.477	0.157	0.091	1.217∗∗∗	−0.739∗∗∗	0.082	0.326∗∗∗	0.573∗∗	0.324	−0.620∗	−0.016
Δ ${POS}_{t-1}$		0.065			3.037∗∗∗	0.180	−0.471∗∗	0.758∗∗						0.410∗∗∗				0.460∗∗∗
Δ ${POS}_{t-2}$		0.218∗∗∗			2.082∗	0.375∗∗		0.076						−0.231∗∗				
Δ ${POS}_{t-3}$		−0.130				−0.695∗∗∗		0.718∗∗										
Δ ${NEG}_{t}$	−0.337	−2.870∗∗∗	0.631	0.648	−2.913	−0.423∗	−0.434	−1.515∗∗	0.281	0.069	0.133	0.422∗∗	0.036	−0.528∗∗∗	−0.124	0.088	0.670	0.650∗∗∗
Δ ${NEG}_{t-1}$		−2.256∗∗∗				0.083							−1.329∗∗∗					0.150
Δ ${NEG}_{t-2}$						−0.743∗∗∗							−2.371∗∗∗					−0.612∗∗∗
Δ ${NEG}_{t-3}$													−1.370∗∗					
ECM(−1)	−0.345	−0.370∗∗∗	−0.587∗∗∗	−0.245∗∗∗	−0.432	−0.061∗∗∗	−0.177	−0.169∗∗∗	−0.291∗∗∗	−0.393	−0.614∗∗∗	−0.452∗∗∗	−0.429∗∗∗	−0.233∗∗∗	−0.554	−0.609	−0.319	−0.116∗∗∗

**Long-term (Panel B)**																		

${GDP}^{KOR}$	−0.548	−4.246∗∗∗	−4.288∗∗∗	−1.773	4.485	7.127	−4.338∗	1.790	−6.217∗	−1.785	−4.410∗∗∗	−2.279∗	−2.279	−2.053∗∗∗	−2.615∗∗	0.186	−1.330	0.361
${GDP}^{F}$	−2.537	2.719	7.647∗∗∗	−0.244	−2.383	0.312	4.021	3.739	9.837∗∗∗	3.288	5.138∗∗∗	1.291∗∗∗	4.519∗∗∗	2.900∗	3.314∗∗	0.126	2.813∗∗∗	1.516
POS	0.897	0.342∗∗∗	0.071	1.323	−1.597	0.921	0.799	−1.056	0.521	0.104	0.767∗	0.089	0.127∗∗	0.663∗∗	0.125	1.042∗∗∗	0.040	1.405∗
NEG	−0.284	−0.555	−0.403	0.363	0.132	4.964	0.284	0.661	1.100	0.204	0.147	0.021	−0.245	−0.088	0.170	0.606∗	1.549∗∗	1.801
Constant	26.143	43.143∗∗	11.951	27.298	−49.199	−107.124	27.972	−53.459	32.083	−1.566	22.802	12.512	−4.455	−11.798	15.082	−4.698	3.236	−21.108

**Diagnostics tests (Panel C)**																		

LM	0.235	0.651	0.146	3.973∗∗	0.666	0.001	0.704	0.312	0.725	0.551	0.135	0.017	3.446∗∗	1.121	1.078	0.259	0.076	0.692
ARCH	0.065	3.020	2.944	5.327∗∗	0.327	0.571	2.734	0.851	0.448	2.301	2.622	1.237	0.008	0.140	5.687∗∗	0.094	0.685	1.073
RESET	1.187	0.082	0.527	4.126∗∗	4.548∗∗	2.469	1.194	0.113	3.275	1.021	0.255	1.140	0.040	0.792	0.159	0.694	0.926	4.954∗∗
CUSUM	S	U	S	U	S	S	S	S	S	S	S	S	S	S	S	S	S	S
${CUSUM}^{2}$	U	S	S	S	U	S	S	U	U	U	U	S	S	US	U	U	U	U

Note: 1) Korea's trading partners are shown as AUS (Australia), AUT (Austria), BEL (Belgium), CAN (Canada), CHE (Switzerland), CHN (China), DEU (Germany), ESP (Spain), FIN (Finland), FRA (France), GBR (United Kingdom), IDN (Indonesia), ITA (Italy), JPN (Japan), NLD (Netherlands), NZL (New Zealand), SGB (Singapore), and USA (United States).

2) POS represents the positive fluctuations and NEG represents the negative fluctuations in the real exchange rate.

3) LM refers to the Breusch-Godfrey serial correlation LM test, ARCH represents the heteroskedasticity test, and RESET is the Ramsey RESET test. In CUSUM and CUSUMQ tests, S and U indicate stable and unstable models, respectively.

4) ∗∗∗, ∗∗, and ∗ denote significance levels of 1 %, 5 %, and 10 %, respectively.

**Table 4:** Results of the FNARDL model.

	Trading partners																	
	AUS	AUT	BEL	CAN	CHE	CHN	DEU	ESP	FIN	FRA	GBR	IDN	ITA	JPN	NLD	NZL	SGB	USA
**Short-term (Panel A)**																		

Δ ${POS}_{t}$	0.475	0.271∗∗∗	0.021	0.533	1.561	0.298∗∗	0.159	0.589∗	−0.081	0.040	1.212∗∗	−0.481∗∗	0.093∗	0.314∗∗∗	0.108	0.301	−0.436	−0.104
Δ ${POS}_{t-1}$					4.484∗∗∗	0.111	−0.370∗							0.455∗∗∗				0.505∗∗∗
Δ ${POS}_{t-2}$					3.226∗∗∗	0.406∗∗∗								−0.282∗∗				0.288∗∗
Δ ${POS}_{t-3}$						−0.521∗∗∗												0.225
Δ ${NEG}_{t}$	0.395	−3.268∗∗∗	0.312	1.726∗∗∗	−1.857	−0.482∗∗	−0.837∗	−1.407∗∗	−0.555	0.496	0.009	0.462∗∗	−0.003	−0.330∗	0.788	0.160	1.068∗	0.398∗
Δ ${NEG}_{t-1}$		−3.859∗∗∗				0.340	−0.471			0.098			−1.961∗∗∗	−0.039				−0.131
Δ ${NEG}_{t-2}$		−1.860∗∗				−0.548∗∗	−1.214∗∗			−1.991∗∗∗			−2.922∗∗∗	0.397∗∗				−0.941∗∗∗
Δ ${NEG}_{t-3}$		−2.145∗∗∗								−1.271∗			−1.799∗∗∗					−0.657∗∗∗
ECM(−1)	−0.474∗∗∗	−0.747∗∗∗	−0.828∗∗∗	−0.729∗∗∗	−0.557∗∗∗	−0.300∗∗∗	−0.430∗∗∗	−0.347∗∗∗	−0.532∗∗∗	−0.602∗∗∗	−0.662∗∗∗	−0.934∗∗∗	−0.386∗∗∗	−0.332∗∗∗	−0.885∗∗∗	−0.663∗∗∗	−0.714∗∗∗	−0.394∗∗∗

**Long-term (Panel B)**																		

${GDP}^{KOR}$	−1.060	−2.862∗∗∗	−3.233∗∗∗	−1.020	−6.412∗	−0.529	2.282	−4.084∗∗	9.798∗∗∗	0.743	−2.284	−1.551∗∗	0.740	−2.019∗∗∗	−0.913	0.180	−2.932∗∗∗	0.526
${GDP}^{F}$	1.102	2.056∗∗	2.842∗∗	0.263	−11.122∗∗	−1.354∗∗∗	−4.968∗	4.284∗∗	1.299	1.377	5.604∗∗∗	1.317∗∗∗	4.211∗∗∗	1.674	5.936∗∗∗	−0.177	2.340∗∗∗	4.377∗∗∗
POS	0.422	0.369∗∗∗	0.128∗∗∗	1.740∗∗∗	−3.023∗	0.645∗∗	−0.309	0.860	−0.004	0.040	1.322∗∗∗	0.125	0.209∗∗	0.543∗∗∗	−0.080	0.930∗∗∗	0.072	−0.187
NEG	0.436	0.023	−0.658∗∗	1.412∗∗∗	−0.264	−0.248	0.450	0.782	0.277	0.833	0.123	0.309∗	1.130	−0.172	1.034∗∗∗	0.369	1.990∗∗∗	0.894∗∗∗
Constant	6.104	27.413∗∗∗	28.447∗∗	12.370	151.067∗∗∗	22.539	10.591	27.544	−155.683∗∗∗	−22.408	−2.284	1.586	−46.097	5.118	−28.362	−2.850	30.202∗∗∗	−52.654∗∗∗

**Fourier terms (Panel C)**																		

$\psi_{1}$	0.035	−0.017	−0.001	−0.177∗∗∗	20.183∗∗∗	−0.104∗∗∗	−0.022	0.170∗∗∗	0.870∗∗∗	−0.070∗	−5.915∗∗∗	−0.064∗∗∗	0.084∗∗∗	−0.005	0.055	0.026	0.870∗∗∗	−0.050∗∗∗
$\psi_{2}$	−0.142∗∗∗	−0.216∗∗∗	−0.189∗∗∗	−0.195∗∗∗	10.644∗∗∗	−0.205∗∗∗	−0.188∗∗∗	−0.021	2.013∗∗∗	−0.154∗∗∗	−6.268∗∗∗	−0.115∗∗∗	−0.043∗∗	−0.030∗∗∗	−0.221∗∗∗	−0.043∗	−0.716∗∗∗	−0.079∗∗∗

**Diagnostics tests (Panel D)**																		

LM	0.005	0.560	1.996	0.230	0.284	0.677	0.532	0.238	0.525	1.735	0.095	0.341	0.221	0.236	0.078	0.296	0.722	0.582
ARCH	0.107	3.701	5.770	2.777	0.070	1.837	2.906	0.239	0.265	0.059	2.305	1.423	0.163	0.152	2.717	0.277	0.053	1.363
RESET	0.000	2.358	0.440	0.784	2.210	0.000	2.735	0.082	2.370	3.340	0.244	6.075∗∗	0.574	0.066	1.017	1.037	2.025	10.898∗∗∗
$W_{SR}$	0.004	42.844∗∗∗	0.131	5.096∗∗	11.900∗∗∗	1.700	3.906∗	4.452∗∗	0.177	2.926∗	1.129	3.479∗	27.279∗∗∗	0.729	0.483	0.029	3.600∗	6.719∗∗∗
$W_{LR}$	0.000	1.791	5.770∗∗	9.334∗∗∗	2.606	7.215∗∗	2.881	0.009	0.167	2.789∗	3.666∗∗	3.601∗	1.120	12.946∗∗∗	6.616∗∗	3.038∗	24.072∗∗∗	10.258∗∗∗
CUSUM	S	S	S	S	S	S	S	S	S	S	S	S	S	S	S	S	S	S
${CUSUM}^{2}$	U	S	S	U	U	S	S	S	S	U	U	S	S	S	U	U	U	U

Note: 1) Korea's trading partners are shown as AUS (Australia), AUT (Austria), BEL (Belgium), CAN (Canada), CHE (Switzerland), CHN (China), DEU (Germany), ESP (Spain), FIN (Finland), FRA (France), GBR (United Kingdom), IDN (Indonesia), ITA (Italy), JPN (Japan), NLD (Netherlands), NZL (New Zealand), SGB (Singapore), and USA (United States).

2) POS represents the positive fluctuations and NEG represents the negative fluctuations in the real exchange rate.

3) LM refers to the Breusch-Godfrey serial correlation LM test, ARCH represents the heteroskedasticity test, and RESET is the Ramsey RESET test. $W_{SR}$ and $W_{LR}$ represent the Wald tests for the short run and long run, respectively. In CUSUM and CUSUMQ tests, S and U indicate stable and unstable models, respectively.

4) ∗∗∗, ∗∗, and ∗ denote significance levels of 1 %, 5 %, and 10 %, respectively.

Table 3 presents the results of the NARDL model. The ΔPOS and ΔNEG (including lags) affected the trade balance in the short run in the cases of AUT, CHE, CHN, DEU, ESP, GBR, IDN, ITA, JPN, NLD, SGB, and USA. Meanwhile, POS (and NEG) had long-term effects only in a few cases such as AUT, GBR, ITA, JPN, NZL, SGB, and the USA. However, the diagnostic tests did not support these results. In particular, the Breusch-Godfrey serial correlation Lagrange multiplier (LM) test did not support the NARDL results for CAN and ITA. Similarly, the heterogeneity test (ARCH test) and the model optimality test (RESET test) did not support the NARDL results for CAN, CHE, NLD, and the USA. Additionally, the CUMSUM or CUMSUM of square (CUSUM^2^) did not provide strong support for the results obtained for AUS, AUT, CAN, CHE, ESP, FIN, FRA, GBR, NLD, NZL, SGB, and the USA.

The results in Panel A of Table 4 reveal that the coefficient of ECM (−1) has a consistently negative sign and is highly significant across all cases. This further corroborates the cointegration results obtained using the F-test in the cointegration-testing step (Table 2). Interestingly, the short-term impact of real exchange rate fluctuations on the trade balance appeared only in a few cases. The coefficient of ΔNEG (including lags) was negative and statistically significant in the case of AUT, CHN, DEU, ESP, FRA, and ITA. By contrast, a strong positive coefficient of ΔNEG was observed for CAN, IDN, and SGB. Moreover, a mixed effect of ΔNEG was observed for JPN and the USA. For ΔPOS (including lag), the effect was strongly positive for AUT, CHE, ESP, GBR, ITA, and the USA, and negative for DEU and IDN. On the other hand, a mixed effect was observed for CHN and JPN. Notably, detecting the impact of real exchange rates (both ΔPOS and ΔNEG) was difficult in the case of AUS, BEL, FIN, NLD, and NZL. Thus, the complex relationship between the real exchange rate and the trade balance is evident. In the short term, Korea's trade balance with certain partners is more negatively than positively influenced by exchange rate fluctuations. In the remaining cases, the J-curve effect is unclear. This is primarily because the essence of the J-curve, interpreted as indicative of short-term deterioration followed by long-term improvement, necessitates a focused examination of the long-term impact of exchange rate fluctuations [45].

Next, we explored the J-curve effect through the results reported in Panel B of Table 4. The long-term impact of changes in the real exchange rate on the trade balance was positive in most cases (11 out of 18 of Korea's trading partners). Although the influence of NEG was insignificant in the case of AUT, CHN, GBR, ITA, JPN, and NZL, $POS^{\prime }s$ coefficient had a positive sign and was statistically significant. This implies that the won's appreciation improved the trade balance. Even though POS was not significant, NEG was positive and significant in the cases of IDN, NLD, SGB, and the USA. This also implies that the won's depreciation stimulates trade balance growth. In the case of CAN, depreciation or appreciation of the won had a positive effect on the trade balance between the two countries, as evidenced by the positive coefficients of both POS and NEG. Nevertheless, the long-term impact of the real exchange rate is unclear for AUS, DEU, ESP, FIN, and FRA. Furthermore, a negative coefficient for POS was found only for CHE, whereas a negative coefficient for NEG was detected only for BEL. Overall, the long-term positive relationship between exchange rate fluctuations and the trade balance between Korea and 11 of its 18 trading partners confirms the strong J-curve pattern.

Finally, to obtain more robust results, diagnostic tests were conducted and the findings are presented in Panel D of Table 4. These tests, including LM, ARCH, and RESET (Ramsey's RESET test), aim to investigate serial correlation, heteroskedasticity, and the model's optimality, respectively. The results show that, at a 5 % significance level, the LM and ARCH test coefficients are not significant in all cases, meaning that the null hypothesis cannot be rejected. This implies the absence of serial correlation and heterogeneity problems in the empirical model. By contrast, the trade balance models of IDN and USA appeared not to be supported by the RESET test. In the remaining cases, RESET's coefficient was not significant, indicating that the models achieved optimality. Next, the asymmetric effects in the short and long run were represented by the W_SR_ and W_LR_ statistics, respectively. W_SR_'s coefficient was significant for AUT, CAN, CHE, DEU, ESP, FRA, IDN, ITA, SGB, and the USA. On the other hand, the results of W_LR_ supported the asymmetric impact of the long-term real exchange rate in 11 cases (BEL, CAN, CHN, FRA, GBR, IDN, JPN, NLD, NZL, SGB, and the USA). Additionally, CUSUM and CUSUM^2^ tests were conducted to assess the stability of the short-term and long-term effects. The results of both the CUSUM and CUSUM^2^ tests suggest stability in the short- and long-term relationships for nine cases: AUT, BEL, CHN, DEU, ESP, FIN, IDN, ITA, and JPN. Although the remaining nine cases were not supported by the CUSUM^2^ test, they were supported by the CUSUM test (see Fig. S1).

Thus, compared to the results in Table 3, the FNARDL model yielded insightful findings regarding the long-term effects of real exchange rates. This was crucial in determining the bilateral J-curve effect in our study. Accordingly, we found that the exchange rate had a positive long-term impact on the trade balances with 11 partners. Nevertheless, such an impact was found in only seven cases using the NARDL model. Furthermore, the diagnostic tests supported the FNARDL model more than the NARDL model. The studies by Refs. [25,27] demonstrated that the J-curve phenomenon has significant policy implications for Korea. In particular, understanding the asymmetric effects of changes in the exchange rate enables the more effective timing of interventions. For instance, during periods of won depreciation, Korean policymakers should prepare for an initial trade deficit. However, these short-term deficits should not deter the implementation of long-term strategies aimed at improving trade balances, such as supporting export-oriented industries and negotiating favorable trade agreements. By anticipating the initial adverse effects and strategically planning for long-term gains, policymakers can better navigate the complexities of international trade and enhance Korea's economic resilience. Therefore, we believe that this study offers valuable information for policymakers.

## 4 Conclusion

This study investigated the bilateral J-curve effect between Korea and 18 trading partners from 1995 Q1 to 2023 Q2. A new approach was applied through the Fourier nonlinear autoregressive distributed lag (FNARDL) model to smooth the data structure. The results of the FNARDL model showed that a long-term positive nonlinear relationship was detected in 11 out of 18 cases. The won's depreciation improved Korea's trade balance with Austria, Canada, China, the UK, Indonesia, Italy, Japan, the Netherlands, New Zealand, Singapore, and the United States. In the remaining cases, the won's depreciation either worsened Korea's trade balance or had an insignificant impact. Meanwhile, the short-term nonlinear impact of the real exchange rate varied depending on each case. Nonetheless, through the Fourier function, the FNARDL model appeared to be better than the NARDL model in identifying the long-term relationship between the exchange rate and the trade balance. The FNARDL model's ability to capture nonlinearities and structural breaks offers a more detailed and accurate understanding of the impact of exchange rate fluctuations on Korea's trade dynamics. This new approach makes a significant methodological contribution to previous empirical studies on this topic in Korea. These findings are crucial for formulating policies that address both immediate economic challenges and long-term strategic goals. Moreover, the current study used bilateral data for 18 of Korea's trading partners, minimizing the problem of aggregation bias, prevalent when using aggregate trade data.

### 4.1 Policy implications

This study's findings have important policy implications, especially for Korea's monetary and trade policy formulation. The long-term positive nonlinear relationship detected in most cases suggests that policymakers should consider the long-term benefits of exchange rate adjustments in improving Korea's trade balance. This finding highlights the importance of strategic exchange rate management and its potential to increase Korea's trade surplus with its major trading partners over time. Furthermore, the variability in the short-term effects across different bilateral relationships underscores the necessity for tailored trade policies that account for the unique dynamics of each trade partnership. This nuanced approach could help mitigate adverse short-term effects while maximizing long-term trade benefits.

The J-curve phenomenon could be elucidated by expanding the scope of the analysis to include more countries and new approaches. This would provide actionable data for policymakers to stabilize Korea's trade balance in the context of exchange rate fluctuations. For example, Korea should promote stronger exports to partners whose trade balances benefit from the won's depreciation. By contrast, it should restrict imports and support exports to partners whose trade balances are disadvantaged by this depreciation.

### 4.2 Research limitations

Despite its comprehensive approach, this study faces limitations that future research should address. The reliance on historical data spanning from 1995 to 2023, while extensive, may not encapsulate the full spectrum of future economic conditions or unforeseen global financial events. Such data constraints may limit the applicability of the findings to future trade dynamics. Moreover, the focus on bilateral trade balances, though insightful, ignores the complex nature of multilateral trade interactions and global economic networks, which could also affect Korea's trade relationships. Moreover, this analysis may not fully account for non-economic factors, such as political changes, trade agreements, and socio-economic shifts that could influence trade flows and exchange rate movements.

### 4.3 Future research directions

Future research could address the limitations of this study and explore several promising avenues. One potential direction is the application of dynamic global trade models that account for multilateral trade relationships and the interconnected nature of the global economy. Further studies could also incorporate more granular data, such as sector-specific trade flows, to uncover how different industries respond to changes in the exchange rate. Moreover, exploring the role of digital trade and services, which are increasingly important in global trade, could provide new insights into the trade balance-exchange rate relationship. Finally, employing predictive analytics and machine-learning techniques may offer novel methodologies for forecasting the effects of exchange rate fluctuations on trade balances.

## CRediT authorship contribution statement

**Min-Joon Kim:** Writing – review & editing, Writing – original draft, Visualization, Validation, Supervision, Software, Methodology, Formal analysis, Conceptualization, Data curation. **Rianmahardhika Sahid Budiharseno:** Writing – original draft, Investigation. **Tong Thanh Trung:** Writing – original draft.

## Declaration of competing interest

The authors declare that they have no known competing financial interests or personal relationships that could have appeared to influence the work reported in this paper.

## Data Availability

Data will be made available based on request.

## References

[1] Doukas J., Lifland S. (1994). Exchange rates and the role of the trade balance account. Manag. Finance.

[2] McKinnon R.I. (1990). The exchange rate and the trade balance: insular versus open economies. Open Econ. Rev..

[3] Bahmani-Oskooee M., Ratha A. (2004). The J-curve dynamics of US bilateral trade. J. Econ. Finance.

[4] Hacker R.S., Hatemi‐J A. (2004). The effect of exchange rate changes on trade balances in the short and long run: evidence from German trade with transitional Central European economies. Econ. Transit..

[5] Narayan P.K., Narayan S. (2004). The J‐curve: evidence from Fiji. Int. Rev. Appl. Econ..

[6] Bahmani‐Oskooee M., Goswami G.G., Kumar Talukdar B. (2005). The bilateral J‐curve: Australia versus her 23 trading partners. Aust. Econ. Pap..

[7] Stucka T. (2004).

[8] Bahmani‐Oskooee M., Hegerty S.W. (2010). The J‐and S‐curves: a survey of the recent literature. J. Econ. Stud..

[9] Wang C.-H., Lin C.-H.A., Yang C.-H. (2012). Short-run and long-run effects of exchange rate change on trade balance: evidence from China and its trading partners. Jpn. World Econ..

[10] Chiloane L., Pretorius M., Botha I. (2014). The relationship between the exchange rate and the trade balance in South Africa. J. Econ. Financ. Stud..

[11] Işik C., Radulescu M., Fedajev A. (2019). The effects of exchange rate depreciations and appreciations on the tourism trade balance: the case of Spain. E. J. Eur. Stud..

[12] ky Chang B. (2005). Changes in effects of exchange rate on trade balance according to industrial structure change. J. Korea Trade.

[13] Hsing H.-M. (2005). Re-Examination of J-curve effect for Japan, Korea and taiwan. Jpn. World Econ..

[14] Bahmani-Oskooee M., Ratha A. (2004). Dynamics of the US trade with developing countries. J. Develop. Area..

[15] Sim S.-H., Chang B.-K. (2006). Bilateral trade balance between Korea and her trading partners: the J-curve effect. J. Korea Trade.

[16] Mustafa M., Rahman M. (2008). US bilateral nominal trade balance with India, Japan, Malaysia, S. Korea and Thailand, and bilateral nominal exchange rate dynamics: evidence on J-curve?. Southwestern Economic Review.

[17] Halicioglu F. (2007). The J‐curve dynamics of Turkish bilateral trade: a cointegration approach. J. Econ. Stud..

[18] Moodley S. (2011).

[19] Bahmani-Oskooee M., Hegerty S.W. (2011). The J-curve and NAFTA: evidence from commodity trade between the US and Mexico. Appl. Econ..

[20] Bao H.H.G., Le H.P. (2021). Asymmetric impact of exchange rate on trade between Vietnam and each of EU-27 countries and the UK: evidence from nonlinear ARDL and the role of vehicle currency. Heliyon.

[21] Baek J. (2013). Does the exchange rate matter to bilateral trade between Korea and Japan? Evidence from commodity trade data. Econ. Modell..

[22] Baek J. (2014). Exchange rate effects on Korea–US bilateral trade: a new look. Res. Econ..

[23] Baek J., Nam S. (2021). The South Korea–China trade and the bilateral real exchange rate: asymmetric evidence from 33 industries. Econ. Anal. Pol..

[24] Baek J., Yoon J.H. (2023). The Korea‐Vietnam trade and the bilateral exchange rate: asymmetric evidence from commodity trade data. Aust. Econ. Pap..

[25] Bahmani-Oskooee M., Xu J., Saha S. (2017). Commodity trade between the US and Korea and the J-curve effect. N. Z. Econ. Pap..

[26] Kim A. (2009). An empirical analysis of Korea's trade imbalances with the US and Japan. J. Asia Pac. Econ..

[27] Bahmani-Oskooee M., Baek J. (2019). Asymmetry cointegration and the J-curve: new evidence from Korean bilateral trade balance models with her 14 partners. J. Asia Pac. Econ..

[28] Cho J.-H. (2019). The real exchange rate effect on bilateral trade balance between Korea and ASEAN countries. Korea Trade Review.

[29] Addai K., Serener B., Kirikkaleli D. (2022). Empirical analysis of the relationship among urbanization, economic growth and ecological footprint: evidence from Eastern Europe. Environ. Sci. Pollut. Control Ser..

[30] Zaghdoudi T., Tissaoui K., Maaloul M.H., Bahou Y., Kammoun N. (2023). Asymmetric connectedness between oil price, coal and renewable energy consumption in China: evidence from Fourier NARDL approach. Energy.

[31] Bahmani-Oskooee M. (1985). Devaluation and the J-curve: some evidence from LDCs. Rev. Econ. Stat..

[32] Bahmani‐Oskooee M., Ardalani Z. (2006). Exchange rate sensitivity of US trade flows: evidence from industry data. South. Econ. J..

[33] Bahmani-Oskooee M., Fariditavana H. (2016). Nonlinear ARDL approach and the J-curve phenomenon. Open Econ. Rev..

[34] Bahmani-Oskooee M., Halicioglu F., Hegerty S.W. (2016). Mexican bilateral trade and the J-curve: an application of the nonlinear ARDL model. Econ. Anal. Pol..

[35] Pesaran M.H., Shin Y., Smith R.J. (2001). Bounds testing approaches to the analysis of level relationships. J. Appl. Econom..

[36] Pal D., Mitra S.K. (2016). Asymmetric oil product pricing in India: evidence from a multiple threshold nonlinear ARDL model. Econ. Modell..

[37] Cheung Y.-W., Chinn M.D., Qian X. (2012). Are Chinese trade flows different?. J. Int. Money Finance.

[38] Kim M.-J., Le T.-T.-H. (2024). Influence analysis of real exchange rate fluctuations on trade balance data using feature important evaluation methods. Information.

[39] Rose A.K. (1991). The role of exchange rates in a popular model of international trade: does the ‘Marshall–Lerner’condition hold?. J. Int. Econ..

[40] Shin Y., Yu B., Greenwood-Nimmo M. (2014). Modelling asymmetric cointegration and dynamic multipliers in a nonlinear ARDL framework. Festschrift in honor of Peter Schmidt: Econometric methods and applications.

[41] Syed Q.R., Apergis N., Goh S.K. (2023). The dynamic relationship between climate policy uncertainty and renewable energy in the US: applying the novel Fourier augmented autoregressive distributed lags approach. Energy.

[42] Kirikkaleli D., Sofuoğlu E., Ojekemi O. (2023). Does patents on environmental technologies matter for the ecological footprint in the USA? Evidence from the novel Fourier ARDL approach. Geosci. Front..

[43] Yilanci V., Bozoklu S., Gorus M.S. (2020). Are BRICS countries pollution havens? Evidence from a bootstrap ARDL bounds testing approach with a Fourier function. Sustain. Cities Soc..

[44] Ludlow J., Enders W. (2000). Estimating non-linear ARMA models using Fourier coefficients. Int. J. Forecast..

[45] Arora S., Bahmani-Oskooee M., Goswami G. (2003). Bilateral J-curve between India and her trading partners. Appl. Econ..

[46] Bahmani-Oskooee M., Brooks T.J. (1999). Bilateral J-curve between US and her trading partners. Weltwirtschaftliches Archiv.

