# Supplementary material for: New insights into Korea's trade relations: A comprehensive FNARDL analysis with 18 global partners
Source: Heliyon. 2024 Oct 22;10(21):e39696. doi: 10.1016/j.heliyon.2024.e39696 (PMC11546184; doi:10.1016/j.heliyon.2024.e39696)
Supplement: Multimedia component 1 [file mmc1.docx]

**Supplementary Information**

**For**

**New Insights into Korea’s Trade Relations: A Comprehensive FNARDL Analysis with 18 Global Partners**

Manuscript submitted to *Heliyon*

October 12^th^, 2024

**Table S1**

List of countries

| **No.** | **Country** | **Alpha-3 code** |
| --- | --- | --- |
| 1 | Australia | AUS |
| 2 | Austria | AUT |
| 3 | Belgium | BEL |
| 4 | Canada | CAN |
| 5 | Switzerland | CHE |
| 6 | China | CHN |
| 7 | Germany | DEU |
| 8 | Spain | ESP |
| 9 | Finland | FIN |
| 10 | France | FRA |
| 11 | United Kingdom | GBR |
| ^12^ | Indonesia | IDN |
| 13 | Italy | ITA |
| 14 | Japan | JPN |
| 15 | Netherlands | NLD |
| 16 | New Zealand | NZL |
| 17 | Singapore | SGB |
| 18 | United States | USA |


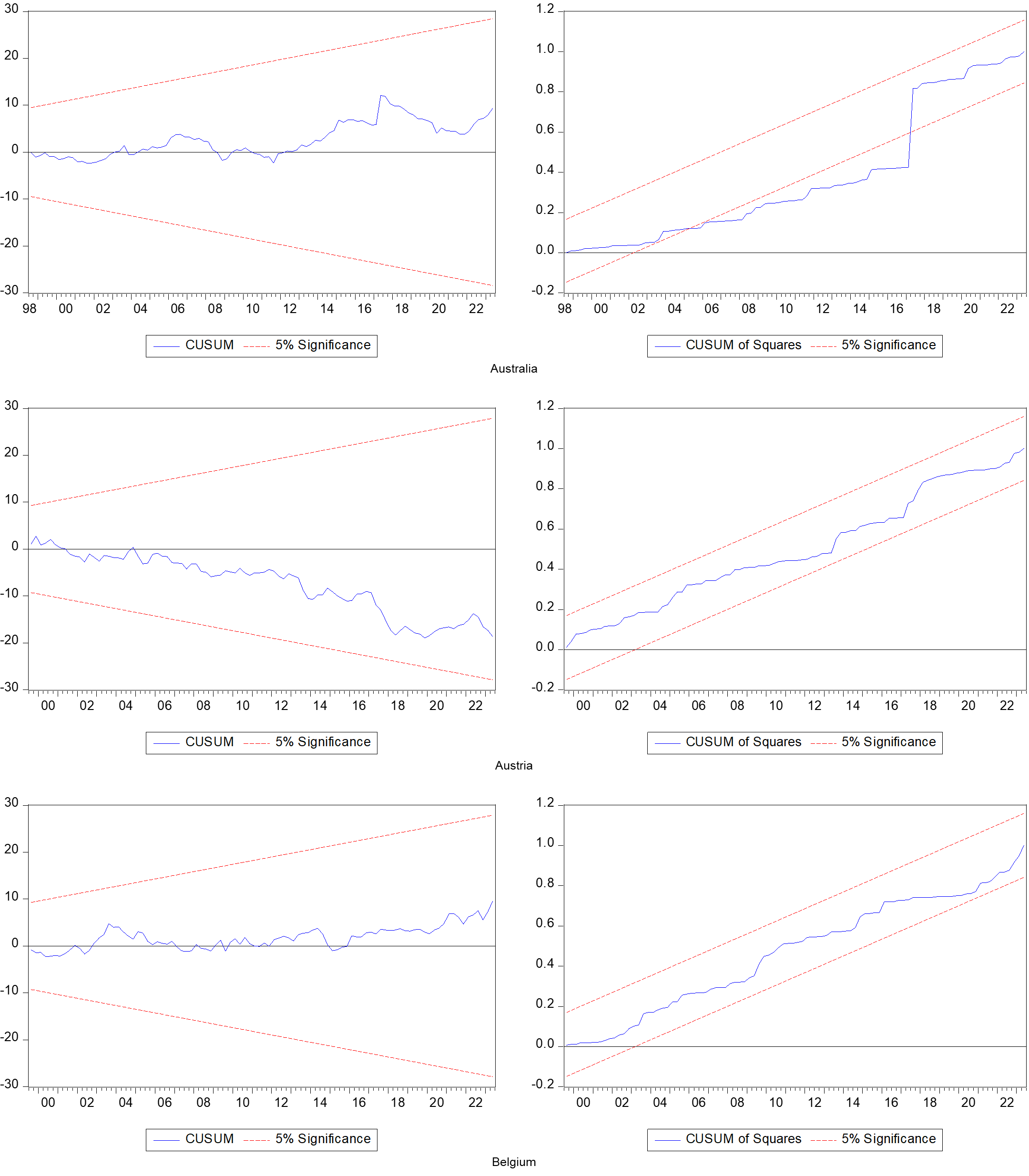


**Fig. S1.** Stability tests for the FNARDL specifications


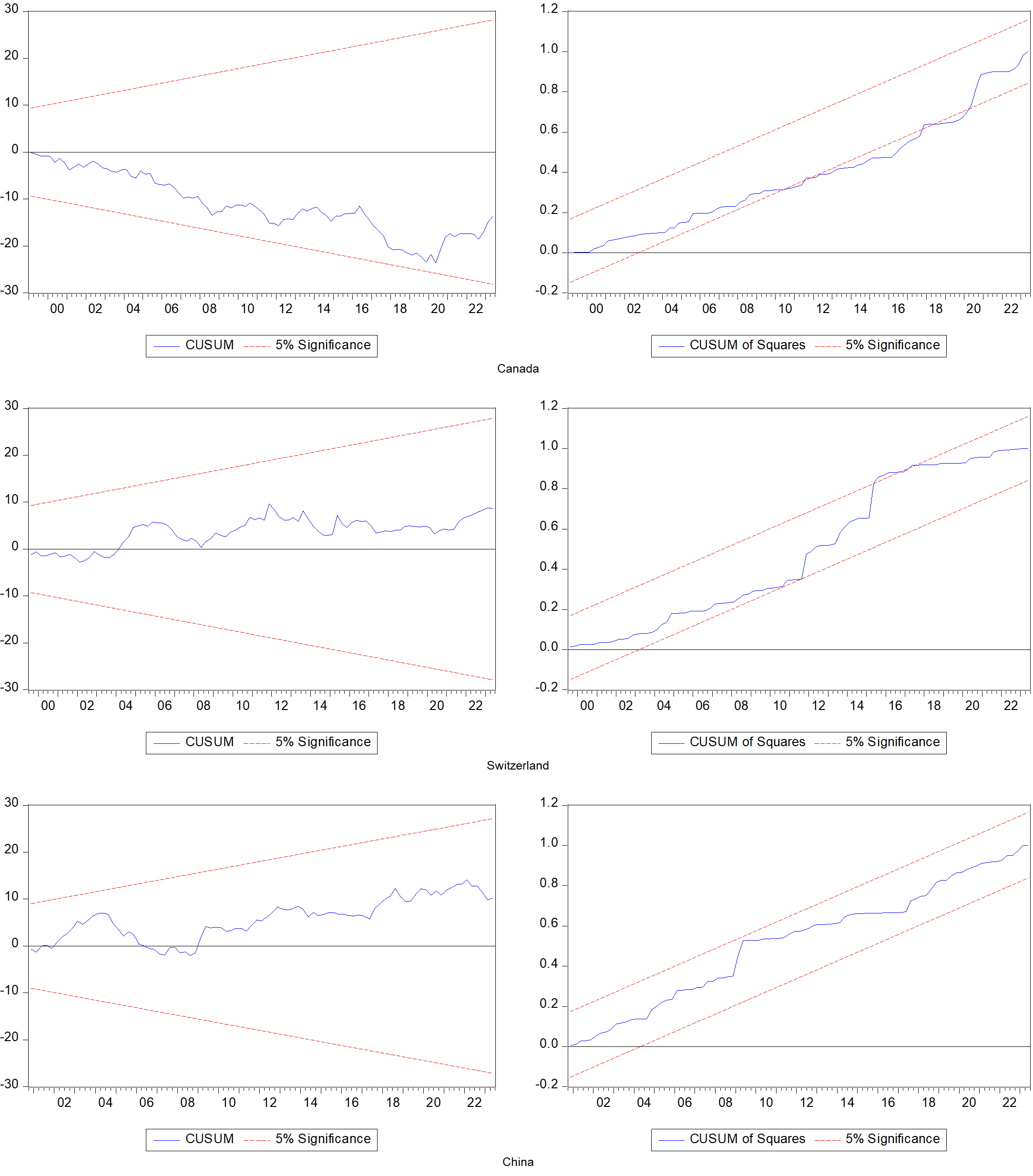


**Fig. S1.** Stability tests for the FNARDL specifications (*Continued*)


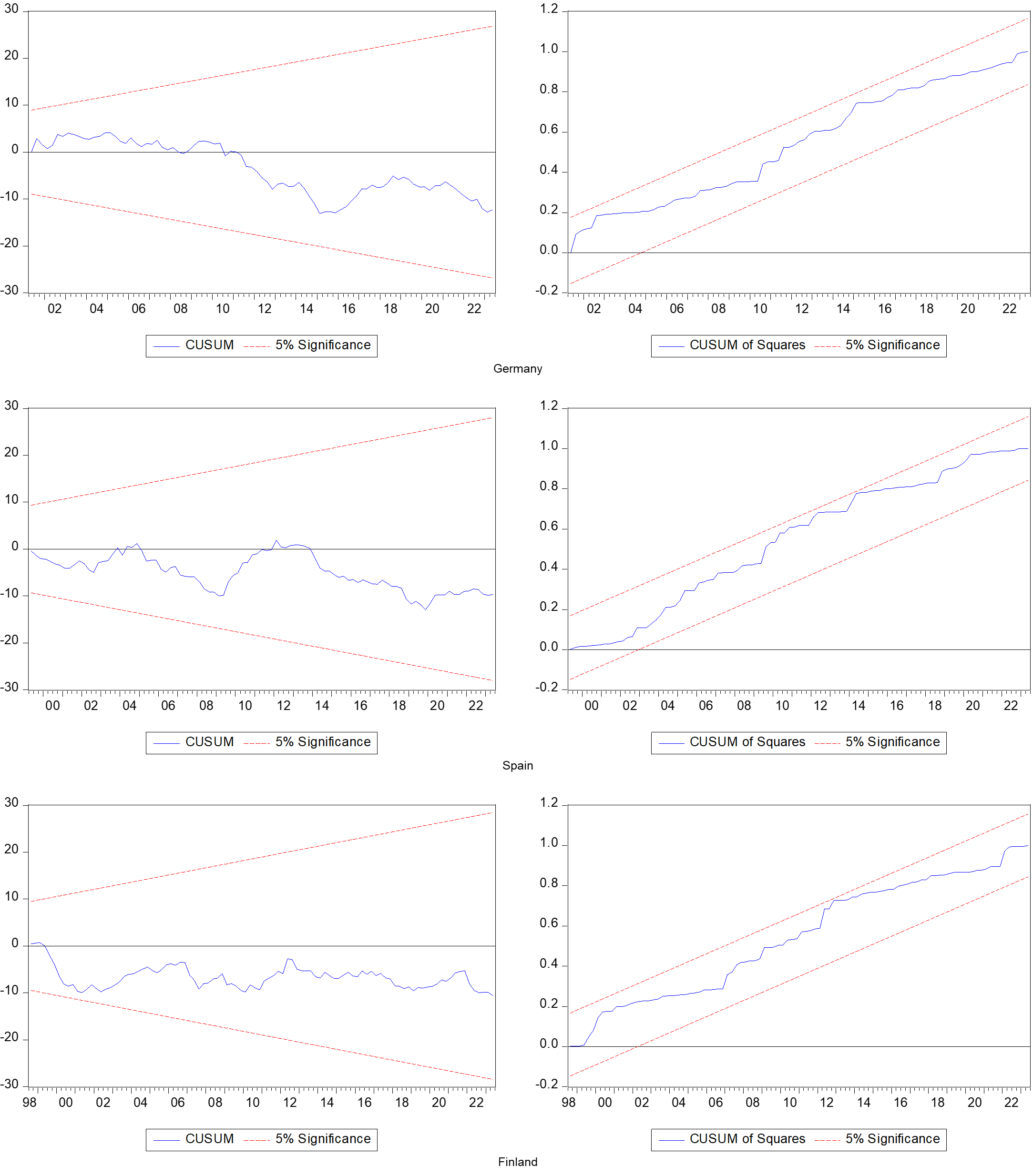


**Fig. S1.** Stability tests for the FNARDL specifications (*Continued*)


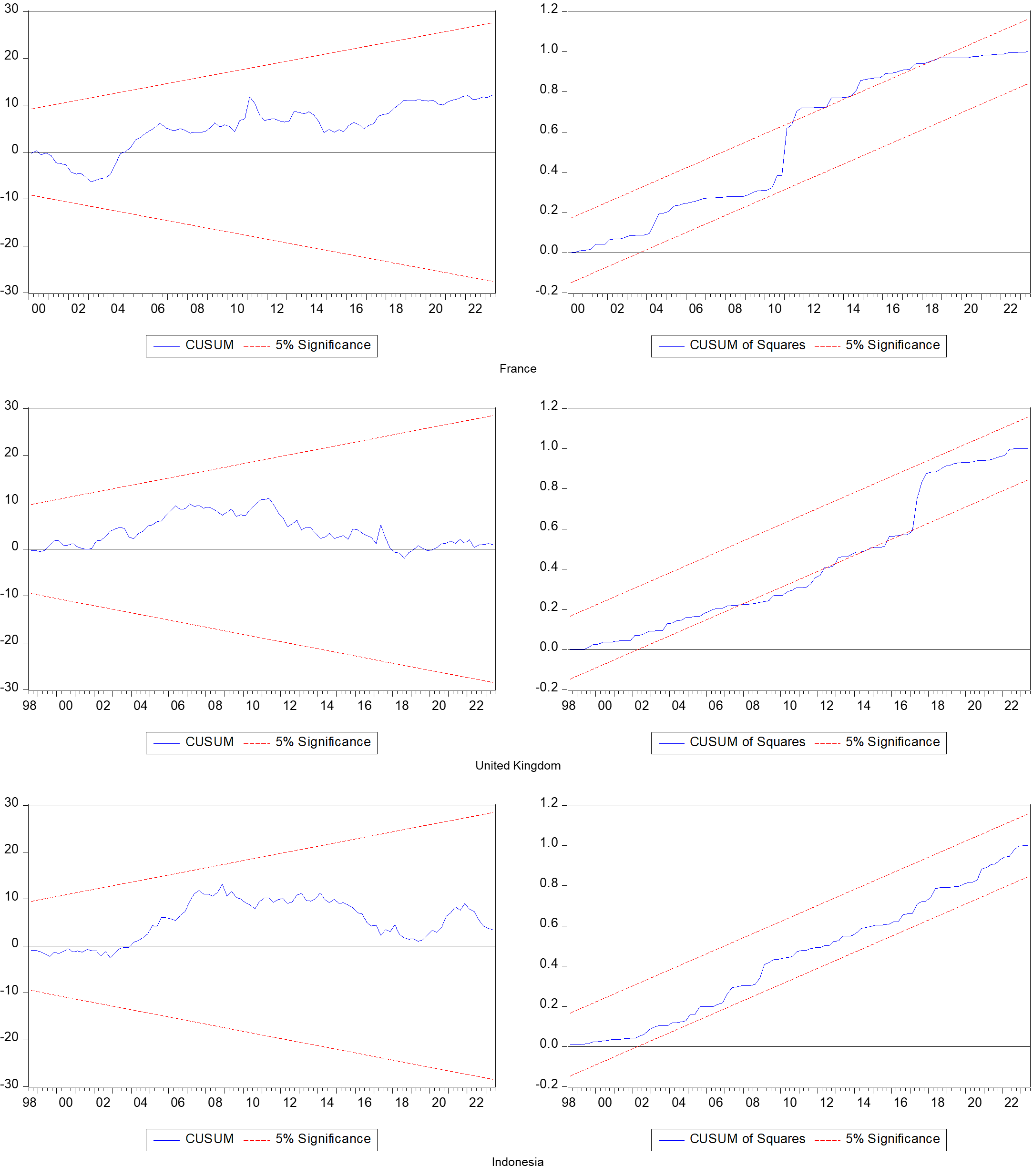


**Fig. S1.** Stability tests for the FNARDL specifications (*Continued*)


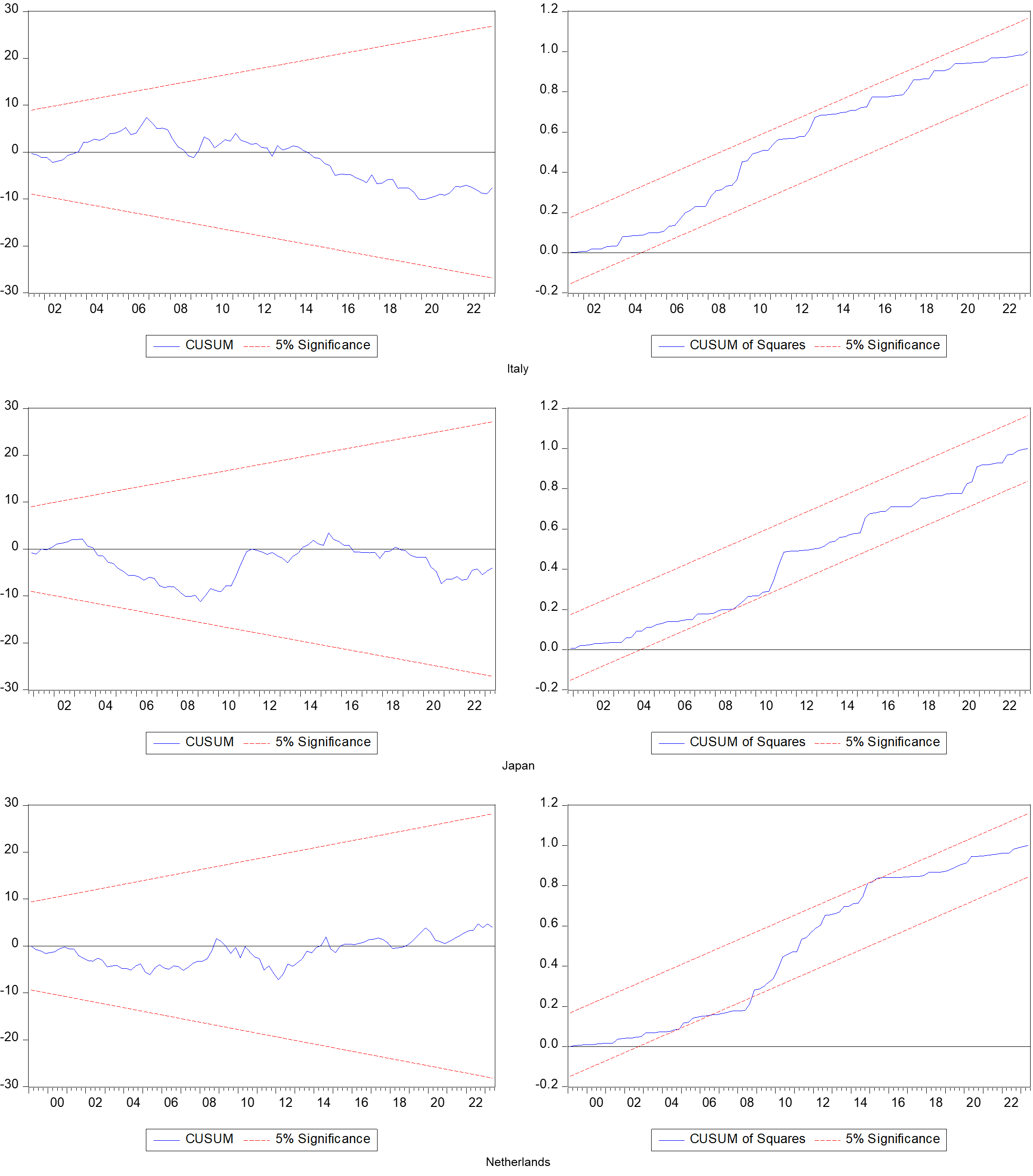


**Fig. S1.** Stability tests for the FNARDL specifications (*Continued*)


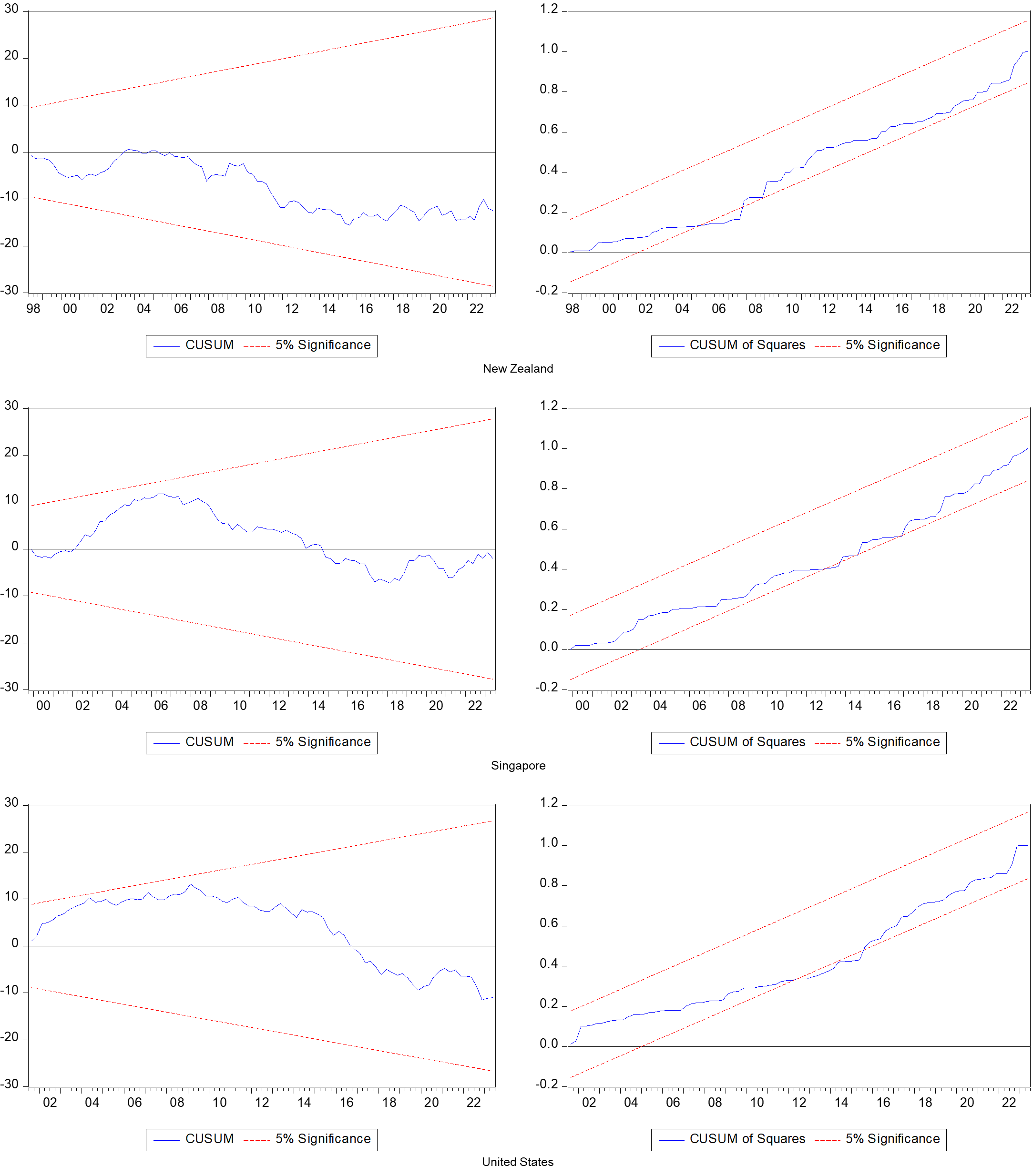


**Fig. S1.** Stability tests for the FNARDL specifications (*Continued*)
